# Basic or Adaptation: The Assessment and Heritability of a Brief Measure of Agency

**DOI:** 10.21203/rs.3.rs-3854555/v1

**Published:** 2024-01-31

**Authors:** Eleanor J. Junkins, D. A. Briley, Jaime Derringer

**Affiliations:** University of Illinois Urbana-Champaign; University of Illinois Urbana-Champaign; University of Illinois Urbana-Champaign

**Keywords:** agency, big five, generativity, longitudinal growth, twin study

## Abstract

The interpersonal circumplex describes two major axes of personality that guide much of social behavior. Agency, one half of the interpersonal circumplex, refers to relatively stable behavioral patterns that center on self-focused dominance and assertiveness. Past empirical work on agency tends to treat the dimension as a characteristic adaptation, rather than a basic component of personality, in part due to the relatively large gender difference in agency with masculine individuals tending to behave more agentic. However, the psychometric overlap between agency and the most closely linked big five dimension, extraversion, is not well-established, and no behavior genetic work has documented evidence concerning the role of genetic and environmental influences. It is unclear whether agency is more similar to a personality trait, with no evidence of shared environmental influence and moderate heritability, or a characteristic adaptation, with some evidence for shared environmental influence and possibly lower heritability. We used the Midlife Development in the United States study to examine agency, big five, and generativity with replication and robustness check (Nnon-twins = 5,194; Ntwins = 1,914; NMilwaukee = 592). Results indicated that agency was higher in men (d = −.24), moderately heritable (44.4%), strongly correlated with extraversion (r = .51), moderately correlated with generativity (r = .36), and that approximately 40% of the variance in agency was shared with the big five. Agency also changed strongly with extraversion and openness, but less so generativity. Altogether, these results indicate that agency functions similar to other basic personality dimensions but is not clearly a dispositional trait.

## Introduction

The interpersonal circumplex (Bakan, 1966; Wiggins, 1979) describes two major axes that guide interpersonal behavior, communion (i.e., getting along) and agency (i.e., getting ahead). Agentic individuals tend to engage socially in a self-focused dominant manner without attention to others. Individuals expect (Eagly & Karau, 2002; Eagly & Wood, 2012) and self-report (Badura et al., 2018; Feingold, 1994; Hsu et al., 2021) higher levels of agency among men. Given evidence for social learning of gender roles (Lott & Maluso, 1993) and the arbitrary nature of many gender-based expectations (Wood & Eagly, 2009), agency may reflect a *characteristic adaptation* (McAdams & Pals, 2006). Alternatively, agency may reflect a *dispositional trait* toward certain thoughts, feelings, or behaviors, similar to other major dimensions of personality like the big five (John et al., 2008). The conceptual distinction between a characteristic adaptation and dispositional trait centers on the role that social learning plays in developing behavioral scripts. For example, individuals may have a dispositional trait toward politeness and respect for others that does not rely on experience or learning, but cultures differ in the sorts of actions that qualify as polite, leading individuals to develop different characteristic adaptations to express their basic level of politeness.

Most previous work has used rational or theoretical approaches to categorize constructs as a dispositional trait or characteristic adaptation. In contrast, Kandler and colleagues (2022) performed biometric decompositions of 50 presumed dispositional traits and 43 presumed characteristic adaptations drawn from the domains of goals, interests, morality, values, and self-schemas. They found that dispositional traits displayed substantially larger evidence for genetic influences, and characteristic adaptations displayed substantially larger environmental influences (correcting for any differences due to measurement error). Here, we test the psychometric and biometric properties of a short measure of agency in the Midlife Development in the United States study. Specifically, we evaluated the phenotypic overlap between agency and measures of dispositional traits (i.e., the big five) and a measure of a characteristic adaptation (i.e., generativity) and test whether the distribution of genetic and environmental effects is more similar to a dispositional trait or characteristic adaptation.

### Nomological Network of Agency

Research domains differ on the conceptualization and measurement of agency (Abele, 2003; Alden et al., 1990; Bem, 2011; Hogan, 1983; McAdams, 1988; Spence & Helmreich, 1979; Wiggins, 1979). A connection to masculinity is common across most research domains. In a meta-analysis of 100,915 participants from 409 studies, Badura and colleagues (2018) found that men score significantly higher on self-report measures of agency than women (*δ* = .48). Importantly, the effect sizes derived to estimate gender differences in agency were based on measures explicitly measuring masculinity (e.g., Bem Sex role Inventory; Personal Attributes Questionnaire), highlighting the explicit conflation of these constructs in the literature. Situating agency within the larger construct space of psychological individual differences can aid interpretation and translation across research domains.

Abele and colleagues (2016) compared a newly constructed Agency-Communion scale to the big five in samples from Germany (*N* = 476), France (*N* = 250), and Australia (*N* = 140). The agency-assertive facet was most strongly associated with extraversion (*r* = .37 – .57) and neuroticism (−.28 – − .56), less strongly with conscientiousness (.19 – .34) and openness (.13 – .27), and not consistently associated with agreeableness (−.2 – .16). Recently it has been suggested that overlap between agency and common measures of the big five might be substantial enough to extract reliable estimates of agency from the big five (Entringer et al., 2021). However, few empirical examples exist in the literature of direct comparisons between the narrow construct of agency and the big five.

Benefits of the big five model of personality are that the factors are identifiable cross-culturally, broadly capture many more narrowly defined facets, and are related to and predictive of many real-world outcomes of interest, such as health, well-being, academic achievement, and job performance (John et al., 2008; Roberts et al., 2007). Further, these associations are highly replicable (Beck & Jackson, 2022; Soto, 2019).

Agency is also related to life outcomes and other indicators of social status. For example, agentic individuals tend to experience more career success (Abele, 2003), higher judgments of status (Fiske et al., 2007), more positive self-perceptions (Wojciszke et al., 2011), and greater achievement (Trapnell & Paulhus, 2012).

From a motivational point of view, generativity (i.e., the inner desire or social expectation to contribute something lasting to the world before death; McAdams & de St. Aubin, 1992) may be a logical link between agency and these outcomes. Highly agentic individuals tend to also express higher levels of generativity (Doerwald et al., 20121). Although generativity is also correlated with the big five (Blatný et al., 2019), generativity shares more conceptual similarity with a characteristic adaptation, rather than a dispositional trait. By situating an explicit measure of agency within a longitudinal study with the big five and a characteristic adaptation, such as generativity, we expand the nomological network of agency.

### Lifespan Trends in Agency

Much is known concerning the development of the big five (Bleidorn et al., 2022; Roberts et al., 2006; Roberts & DelVecchio, 2000). Personality development continues throughout the lifespan, with mean-levels shifting about a standard deviation in more “mature” directions with increasing age (i.e., the maturity principle). This change is concentrated in late adolescence and early adulthood (Roberts & Davis, 2016). Social dominance, conceptually similar to agency, increased substantially across the lifespan, with most of the increases occurring in early adulthood. However, it is not entirely clear whether social dominance is an appropriate marker for agency. For example, Diehl and colleagues (2004) found that younger adults were much more likely to self-attribute agency descriptors (*r* = − .49) and agency self-attributions decrease across age. Given that Roberts et al. (2006)’s meta-analytic evidence was based on longitudinal studies which estimated within-person change, the discrepancy may be due to cohort effects.

Test-retest stability increases across the lifespan (i.e., cumulative continuity principle; Bleidorn et al., 2022). Roberts & DelVecchio (2000) synthesized longitudinal studies of personality, including masculinity/femininity (*k* = 17). Results were consistent with moderate-to-strong rank-order stability across time, particularly in adulthood. Stability was not substantially different between measures of the big five and masculinity/femininity. Further, McAdams et al. (2006) found 3-year test-retest stability of .43 for agency derived from narrative interviews which draw more heavily on the motivation and identity components of agency. We extend these previous studies by tracking stability and change of agency across two decades of midlife development.

### Behavior Genetic Studies of Agency

Genetic influences on broad personality domains are well-established. Meta-analytic estimates tend to converge on approximately 40–60% of the variance in personality being associated with genotypic variation (Vukosovic & Bratko, 2015), with little evidence of differential heritability across domains or levels of the trait hierarchy (Turkhiemer et al., 2014). Individual differences in change and rank-order stability are both due in part to genetic influences (Bleidorn et al., 2010; Briley & Tucker-Drob, 2014; Kandler & Papendick, 2017). The heritability of various masculine, or dominant, traits have been estimated between 24–60% (see Supplement Table S1; Bailey et al., 2000; Gottesman, 1966; Hopwood et al., 2011; Lippa & Hershberger, 1999; McCartney et al., 1990; Mitchell et al., 1989). The lower end of these estimates is similar to what Kandler et al. (2022) found for characteristic adaptations, with the higher end more consistent with results for dispositional traits. This ambiguity leaves unaddressed whether agency is better thought of as a characteristic adaptation or dispositional trait. To the best of our knowledge, no direct biometric estimates of agency have been reported. Here, we provide an estimate of heritability for agency, as well as estimates for stability and individual differences in change, and compare the magnitude of this value to that found for dispositional traits and characteristics adaptations (Kandler et al., 2022).

### The Current Study

We sought to assess the psychometric and biometric features of a brief measure of agency available from the National Survey of Midlife Development in the United States (MIDUS; Brim et al., 1999) using three waves of longitudinal data. We do so by analyzing gender differences, measurement invariance, associations with generativity, and relationship to the big five personality traits among unrelated individuals cross-sectionally and longitudinally (*N* = 5,194). We then demonstrate consistency of the results using the twin subsample and estimate genetic and environmental (co)variance (*N* = 1,914). The present research tested whether agency conforms to theoretical expectations of a characteristic adaptation or dispositional trait by addressing its relationships to gender, the big five, and generativity within and across time.

## Methods

### Participants

#### Non-twin Sample

The first wave of the National Survey of Midlife Development in the United States (MIDUS 1) collected a general population sample from 1995–1996 yielding 7,108 participants. The collaborative project investigated patterns, predictors, and consequences of midlife development in terms of physical health, psychological well-being, and social responsibility. The full sample consisted of unrelated individuals, siblings, and twins (Brim et al., 1999). We used all available non-twins for the first set of phenotypic analyses, totaling 5,194 participants with mean age 46.93 years (range from 20 to 75, *SD* = 13.27 years). The sample was 50.2% female and 49.8% male. The self-reported races of participants were White (*N* = 4,016), Black and/or African American (*N* = 255), Native American or Aleutian Islander (*N* = 28), Asian or Pacific Islander (*N* = 60), Multiracial (*N* = 40), and 106 identified as “other”. Study variables were chosen for their expected associations with Agency. For a negative control (i.e., showing not everything is correlated) we included BMI of the participants pre-calculated in the MIDUS data in the correlation matrices.

Participants were followed longitudinally with a time lag between assessments of roughly a decade. Response rates for phone interviews from wave 1 to wave 2 were 75%[1] and from wave 2 to wave 3 were 77%. The self-administered questionnaires yielded a total of 3,479 at wave 2 (52.3% female and 47.7% male) and 2,276 at wave 3 (53.3% female and 46.7% male). The mean age for the at wave 2 was 56.01 years (range from 28 to 84, SD = 12.72 years) and 64.17 years (range from 39 to 93, SD = 11.57 years) at wave 3. The wave 3 non-twin sample had low racial diversity (White/Caucasian = 2,012; Black and/or African American = 85; Asian or Pacific Islander = 16; Multiracial = 22; Other and Native American or Aleutian Islander = 41). All data used in this report are publicly available[2].

#### Twin Sample

Next, we used the twin sample to decompose variance in the phenotypes into genetic and environmental components. There was a total of 1,914 individuals (31 missing zygosity information), and the sample was 55.3% female and 44.7% male. The self-reported race of the participants was as follows: White (*N* = 1,632), Black and/or African American (76), Native American or Aleutian Islander/Eskimo (11), Multiracial (12), and 18 identified as “other”. The twin sample had mean age 44.89 years (range from 25 to 75, *SD* = 12.07 years). Means, standard deviations, and Cronbach’s alpha reliabilities in the twin sample are reported in Table 1.

Zygosity was determined via an eight-item self-report screener which asked about physical similarity. Although zygosity classification was not verified with genotyping, similar studies using the same items have found accuracy to be over 90% when confirmed via genotyping (Lykken et al., 1990). Triplets were included pairwise and weighted to correct for the same individual appearing in multiple pairs. The resulting twin sample included 347 MZ pairs, 322 DZ pairs, and 252 DZOS pairs (total = 921 pairs). The gender breakdown for same-sex pairs was 185 MZF, 162 MZM, 200 DZF, and 122 DZM. At the final wave, there were 107 MZF, 86 MZM, 109 DZF, 53 DZM and 145 DZOS pairs.

#### Milwaukee Sample

In order to better examine health issues in minority populations, the Midlife in the United States study sampled Milwaukee, Wisconsin based on areas of the city with high concentrations of African American residents. The cross-time correlations will be reported as a robustness check of the generalizability of the cross-time correlations in non-twins and twins.

### Measures

#### Personality Traits

Personality was measured by the Midlife Development Inventory (MIDI; Lachman & Weaver, 1997). The MIDI personality inventory contains 30 adjectives that assess neuroticism, conscientiousness, extraversion, agreeableness, openness to experience, and agency. Each trait is measured by 4 to 7 items. Participants rated items on a 4-point scale indicating whether the adjective described them “not at all” to “a lot.” Means, standard deviations, and Cronbach’s alpha reliability for the items and scales are reported in Table S2.

#### Loyola Generativity Scale- contributions domain

Participants completed six items rated on a 4-point scale from the Loyola Generativity Scale (McAdams & de St. Aubin, 1992) to measure the specific generativity domain of “contributions.” Example items were: “Many people come to you for advice” and “You have had a good influence on the lives of many people.” Psychometric properties of this scale are reported in Table S2.

### Statistical Analyses

Analytic scripts are available at the OSF link: https://osf.io/qsj9u/? view_only=5794f5d6b414474a953000337de38b95. Analyses were conducted using R (R Core Team, 2022) and the packages *lavaan*, *semTools*, *effsize*, *uMx*, and *psych* (Bates et al., 2019; Jorgensen et al., 2021; Revelle, 2020; Rosseel, 2012; Torchiano, 2020). Given the large sample size and aims of the current study, we focus on effect size estimates and precision throughout. To correct for non-independence of observations due to the familial structure of the data, we used cluster-robust standard errors (McNeish & Harring, 2017). All models use full information maximum likelihood estimation to handle missing data.

### Measurement Invariance

Measurement invariance was examined between men and women and across time by testing the thresholds, loadings, and intercepts for equivalence (Svetina et al., 2020; Wu & Estabrook, 2016). We used standard cutoffs for determining whether the assumptions of measurement invariance hold (i.e.,ΔCFI≤ 0.01). Model comparisons indicated that factor scaling was consistent across sex and time (see Table S3, S6, and S7 for complete details). After establishing measurement invariance, subsequent analyses were carried out with the mean scores.

### Attrition Analysis

We estimated Cohen’s *d* effect sizes comparing baseline scores for those who remained in the study compared to those who did not participate at the second or third wave. All differences were small (|*d*| < .21; see Table S4).

### Phenotypic Analyses

We estimated bivariate correlations and multiple regressions between agency and the other phenotypes cross-sectionally. These analyses indicate the extent to which agency shares common variance with dispositional traits and the characteristic adaptation of generativity. To estimate longitudinal trends, we used linear growth curve models and correlated intercepts and slopes across phenotypes. Substantial variance in slopes indicates that individuals follow varying trajectories of development. Correlated slopes indicate that individual differences in change in one construct overlap with individual differences in change in another construct. Put differently, if the extent to which someone changes in agency is similar to their change in generativity, a positive slope-slope correlation would be found. Such models can estimate the extent to which the phenotypes co-develop, possibly because one phenotype causally shapes the development of the other, reciprocal causal processes, or some other variable causally impacts the development of both phenotypes.

### Behavior Genetic Analyses

We decomposed the variance in each phenotype using the genetically informative twin subsample (Neale & Cardon, 1992). Because MZ twins are more genetically similar than DZ twins, larger MZ twin correlations compared to DZ twin correlations indicate additive genetic influences (A). If twins are more psychologically similar than would be expected due to additive genetic influences alone, this result indicates that shared environmental factors (C) also influence the phenotype. However, if MZ twin similarity is more than double DZ twin similarity, this result indicates that dominant genetic influences (D) are plausible. The classical twin design does not provide sufficient information to identify C and D simultaneously, and therefore are not estimated in the same model. Finally, unless MZ twins are psychologically identical, nonshared environmental influences (E) lead to differences between twins.

We formalized the assumptions of this model in a structural equation modeling framework where variance in the phenotypes was decomposed into A, C, D, and E factors, depending on the pattern of twin correlations. For MZ twins, the correlation between factors representing genetic and shared environmental influences were fixed to 1, reflecting that MZ twins share nearly identical genotypes and shared environmental influences. For DZ twins, the correlation between the A factors were fixed to .5, reflecting the assumption that DZ twins share, on average, 50% of segregating genetic material. The correlation between D factors was fixed to .25, reflecting the probability that the twins share the dominant allele. The correlation between C factors was fixed to 1 as all siblings, by definition, have the same shared environment. For all twin pairs, the correlation between E factors was fixed to 0 due to these effects being individual-specific. Multivariate extensions of these models are premised on the same logic and allow for decomposing covariance between phenotypes into genetic and environmental components. Because we included opposite-sex pairs, all phenotypes were residualized for age and sex, as is standard practice (McGue & Bouchard, 1984).

Using these behavior genetic techniques, we estimated a series of univariate models to identify the best fitting set of variance components to represent each phenotype (see Table S10 for cross-twin cross-trait correlations). These analyses indicate the extent to which genetic and environmental influences contribute to the development of agency. Then, we estimated bivariate models to decompose the covariance between agency and the other phenotypes. These analyses indicate the extent to which genetic and environmental influences link agency with dispositional traits and a characteristic adaptation. We used a multivariate Cholesky decomposition, the behavior genetic analogue of multiple regression, to estimate associations with agency controlling for the other included phenotypes. In contrast to regression, the order in which phenotypes are entered into the model impacts interpretation. We specified phenotypes with the weakest association with agency to take precedent in the model to limit convergence issues. Finally, we decomposed the (co)variance of intercepts and slopes of growth curves for each pair of phenotypes to evaluate the extent to which genetic and environmental influences guide individual differences in trajectories of development. In one instance, we encountered convergence difficulties when decomposing the intercept and slope latent factors. To provide estimates, we performed the decomposition on factor scores derived from the phenotypic model.

## Results

### Descriptive Statistics

After establishing measurement invariance, we calculated descriptive statistics (Table 1) and bivariate correlations (Table 2) split by gender for variables standardized with respect to the baseline mean and standard deviation. Item-level descriptive statists are reported in Table S1. Because descriptive information concerning the big five have been reported elsewhere using these data (Graham et al., 2020; Olaru & Allemand, 2022), instead we focus on agency, generativity, and their links to the big five.

At baseline, men reported modestly higher levels of agency than women in the non-twin (*d* = −0.24, 99%CI [−0.317, −0.162]) and twin samples (*d* = −0.24, 99%CI [−0.368, −0.116]; see Figure S1–S3 for distributions and item information curves). Women were modestly more variable in agency relative to men. Despite typical descriptions of agency centering on masculinity, distributions of agency were largely overlapping. Generativity showed even smaller gender differences in mean and variance. Results were largely similar across waves (Table S5).

Consistent with expectations, agency was moderately-to-strongly correlated with extraversion and moderately correlated with generativity. Openness was also moderately-to-strongly correlated with agency. Agentic individuals reported being outgoing, active, imaginative, creative, and desiring to leave a mark on the world to a greater extent than less agentic individuals. Generative individuals also reported higher levels of extraversion and openness, similar to agentic individuals. In contrast, generative individuals reported higher levels of agreeableness. This association was much weaker for agency, indicating that being warm, caring, and sympathetic are personological distinguishing factors between agency and generativity. Associations with the other personality dimensions were more modest. Age was uncorrelated with Agency (−0.012).

Multiple regression results (Table 3) further supported agreeableness as playing a differential role for agency and generativity. For both phenotypes, extraversion and openness retained moderate, positive associations even when controlling for the other big five. For agency, agreeableness was estimated to have a negative association when controlling for the big five. Put differently, among individuals with similar levels of extraversion and openness, more agreeable individuals would be expected to have lower levels of agency. At the zero-order level, this negative association was masked due to the positive associations among agreeableness, extraversion, and openness. Overall, approximately 40% of the variance in agency was associated with the big five. Thus, although agency and generativity share variance with the big five, neither phenotype primarily reflects typical dispositional traits.

### Do the phenotypes display co-development?

Next, we evaluated the extent to which the longitudinal development of agency and generativity were associated with trajectories of big five development. All models fit the data well (see Table S8–S9 for complete parameter estimates and fit statistics). On average, agency decreased slightly across time (−.326 baseline-SD units), but there was considerable variance in trajectories (slope variance = .031). Put differently, these results imply that someone following a more positive (i.e., 1.5 slope SDs above the mean) or more negative (i.e., 1.5 slope SDs below the mean) would show − .062 and − .590 baseline-SD units of change in agency across waves.

Individual differences in change for agency were weakly-to-modestly related to baseline levels of the big five (Table 4). The largest associations were with extraversion and openness, both of which were negative. These results indicate that individuals who began the study relatively higher on extraversion and openness tended to display smaller increases or larger decreases in agency across time. Correlations among slopes were much stronger. Individuals who followed more positive trajectories of growth in extraversion (*r* = .90) and openness (*r* = .93) also followed very similar trajectories of agency development, relative to other participants in the sample. Trajectories of agreeableness (*r* = .49) and conscientiousness (*r* = .50) were also moderately correlated with trajectories of agency.

Results for generativity showed generatively only moderately changed with agency. Starting levels of either generativity or agency were weakly correlated with change in the other. Despite stronger within-time correlations, agency changed more positively with agreeableness or conscientiousness than generativity.

Taken together, these results imply that agency codevelops along with dispositional traits like the big five, with nearly identical relative trajectories among agency, extraversion, and openness. Co-development was weaker with generativity, a characteristic adaptation.

### How heritable are levels and trajectories of agency, generativity, and the big five?

In the supplemental materials (Tables S11-S16), we report time-specific behavior genetic decompositions, as well as multivariate extensions. Broadly, these results indicate that agency is moderately heritable (44.4%), that shared variance with dispositional traits tended to reflect a mix of primarily genetic and some nonshared environmental sources, and that residual variance in agency was primarily nonshared environmental (including measurement error) with modest evidence of unique genetic effects (~ 5% of the total variance). Generativity, on the other hand, was estimated 32.4% due to genetic contribution in line with Kandler and colleagues’ (2022) findings Behavior genetic results for the big five in these data have been reported in greater detail elsewhere (Luo et al., 2017; South et al., 2018). Here, we focus on results derived from growth curve models.

We estimated the heritability and environmentality of the intercept and slope factors for each phenotype (Table S18–S19). For the big five, the heritability of the intercept and slope factors ranged from 44.4–70.9% for the intercepts and 0–62.9% for the slopes. Genetic influences accounted for 63.7% and 65.8% of the variance in the intercept and slope of agency, respectively. Results for generativity showed much lower genetic influences for the intercept, 39.6%, and no genetic influences for the slope factor.

Finally, we decomposed the estimated phenotypic correlations among intercepts and slopes into genetic and environmental contributions. The genetic contribution to a phenotypic correlation indicates the extent to which the correlation is due to genetic sources of variance, in correlation units. For example, a phenotypic correlation of .8 could be decomposed into a genetic contribution of .6 and a nonshared environmental contribution of .2, which sums to the phenotypic correlation of .8. These statistics can be calculated from the heritability, environmentality, and genetic and environmental correlations for the phenotypes (see Briley & Tucker-Drob, 2014 for further details).

Table 5 reports genetic and environmental contributions to correlations across intercepts and slopes among the phenotypes. Focusing first on the intercepts of agency and generativity, the genetic correlation was .416 and environmental correlation was .316 which was in line with the phenotypic correlations being third largest. In terms of the slopes of agency and generativity, there was correlated shared environment suggesting that living in environments that function to make people more similar on agency are partially overlapping with environments that shape people on generativity. Extraversion and openness were also strongly correlated genetically and environmentally with agency intercept and slope. The starting level of extraversion was strongly genetically correlated with the starting level of agency as well as the change in agency. The starting level of openness was strongly genetically correlated with the starting level of agency and weakly genetically correlated with the slope of agency. Which is to say that the starting levels of dispositional traits were strongly genetically related to starting levels of agency. Change in agency was strongly genetically related to change in extraversion and shared environmentally correlated with generativity.

## Discussion

Personality psychologists have relied on the distinction between dispositional traits and characteristic adaptations to distinguish aspects of persons that develop largely without or with contextual inputs, respectively. Following Kandler et al. (2022), we used genetically informative data to test whether agency, a personality attribute centered on assertiveness and self-confidence in interpersonal relations, is more similar to a dispositional trait, like the big five, or a characteristic adaptation, like generativity. Taken as a whole, the results were somewhat ambiguous. Agency was moderately-to-strongly correlated with the big five and displayed nearly identical relative growth trajectories compared to extraversion and openness. These results were found in non-twins, replicated in twins, and cross-time correlations of the Milwaukee oversample all converged on similar findings. Further, the magnitude of genetic and environmental influences was similar and the covariance between trajectories of agency and the big five were disproportionately due to genetic sources of variance. Each of these pieces of evidence would point to the construct of agency operating as a dispositional trait. However, many of these pieces of evidence were also found for generativity, a construct that much more clearly meets the definition of a characteristic adaptation. As with many binary categorization systems (e.g., Morgenroth & Ryan, 2020), this deceptively simple distinction may hide an underlying continuous dimension of input from external sources.

Early descriptions of the big five in terms of dispositional traits were explicit and clear that the big five were unable to be altered by the environment (e.g., McCrae & Costa Jr., 1999), but as evidence inconsistent with such strong claims accumulated (e.g., Bleidorn et al., 2013; Bühler et al., 2023; Dugan et al., 2023; Gurven et al., 2013; Hudson & Fraley, 2017; Niehoff et al., 2017; Obschonka et al., 2018; Roberts et al., 2017; Tucker-Drob & Briley, 2014), the distinction became fuzzy (e.g., McCrae & Costa Jr., 2008). It seems obvious that some aspects of human development are more difficult to change than others. Height may be quite difficult to actively control given adequate nutrition, whereas short-term states such as low positive affect could be actively raised through socializing or exercising. Highly stable constructs, such as cognitive ability, may at first glance seem to reflect a dispositional trait, but education is a volitional action that causally raises general ability (Ritchie & Tucker-Drob, 2018). Rather than attempting to fit constructs and messy developmental processes into neat categories, it may make more sense to collect a range of psychometric, developmental, and behavioral aspects of constructs in order to place them along a continuum. Importantly, it is not clear what sorts of properties would be necessary and sufficient to identify this continuum. Heritability estimates have been used, improperly, to justify claims about insensitivity to environmental input. However, heritability estimates include gene-environment interplay processes which could lead to high heritability estimates with many modifiable environmental risk factors, or novel interventions could be designed to work in tandem with genetically influenced characteristics (e.g., wearing eyeglasses). We favor the approach taken by Kandler et al. (2022) which uses behavior genetic estimates as one data point among many to guide inferences. In this context, agency appears to reflect an intermediate variable.

### Limitations and Future Directions

Our study was the first investigation of the heritability of agency in MIDUS. Only one recent study was found on the genetic and environmental correlations of personality and loneliness that included agency (Freilich et al., 2022). However, our focus was on the correlates of agency cross-sectionally and longitudinally and they were interested in loneliness and personality correlates cross-sectionally. One study was found to study agency in MIDUS longitudinally with well-being (Haas & vanDellen, 2020) but not among personality traits.

One limitation was the available measures of agency and the big five. The MIDI personality scales are an extra short personality inventory that is not often used by personality researchers. Because of its inclusion in a wide-ranging population study, the MIDI inventory necessarily values brevity over breadth; therefore, the question of whether agency encompasses specific facets under such traits as extraversion and openness or whether agency explains the differences between men and women on the big five traits remains unanswered. The current measure, also, lacked a distinct communion scale. That being said, even the brief measure of agency showed results that aligned with expectations and falls somewhere between a dispositional trait and characteristic adaptation.

The second limitation was lack of generalizability across cohorts, race, and gender diverse people. The current study, which used cohorts who were born 1920 to 1975, was not equipped to test for cohort effects we would expect to see in younger cohorts or those born nearer the turn of the 21st century. Whether gender differences in agency remain stable over generations and whether the pattern of associations persist from one generation to the next are two questions that would be useful to test with more recent cohorts. Next, although the sample was collected to be population representative, racial diversity was still insufficient to provide appropriate sample sizes to specifically test equality of effects sizes across races or ethnicities. Finally, there were no gender diverse people included in MIDUS by its reporting of respondent’s sex as “male”, “female”, or missing. Not even allowing a third option leads to explicit exclusion of certain groups.

Lastly, three waves ten years apart are available in MIDUS and so our analysis will not be very sensitive to change. Instead, growth over long time lags alludes to broader abstraction of how personality changes from one decade of life to the next. Specific mechanisms of change are not captured with our design.

### Future Research

To understand the relationships of agency and related constructs better, future research should examine a more robust measure of agency, and ideally communion, in longitudinal data with larger diversity of sampled race, ethnicity, and gender in younger cohorts. Refined measurement of agency-communion values shows promise in motives research (e.g., Conroy & Green, 2020), political psychology (e.g., Beattie et al., 2019), and social isolation (e.g., Helm et al., 2018). Agency-communion traits similarly has been illuminating for stereotyping (e.g., Klysing et al., 2021), interpersonal perception (e.g., Abele & Yzerbyt, 2021), and hiring practices/discrimination and workplace behaviors (e.g., Chalmers, 2021; Kahalon et al., 2021). This being only a small selection of the current research underway with agency-communion.

### General Conclusions

Agency was most strongly correlated with openness to experience, extraversion, and generativity. Change in agency was most strongly related to change in extraversion and openness and less related to change in generativity. Across both samples, we observed a consistent but small gender difference in agency, which was not accounted for by differences in measurement properties. In the twin sample, agency demonstrated similar heritability to what is reported for other personality traits (around 40%; Vukasović & Bratko, 2015). Our studies serve as an evaluation of the brief measure of agency and whether it fits a dispositional trait, like the big five, or characteristic adaptation, like generativity. We have shown these associations longitudinally in a genetically-informative, large, national U.S. sample. We suggest these results be used as a pilot study and replicated in unique samples with more refined trait measures of agency-communion (e.g., Abele et al., 2016) considerate of all gender identities. We should also consider that there is likely a continuous distribution of variables that fall more in line with dispositions versus adaptations.

## Figures and Tables

**Table 1:** Descriptive statistics for non-twin (*N* = 5,194) and twin samples (*N* = 1,914).

	Women (N = 2,607; 1059)						Men (N = 2,585; 855)					
	Non-twin			Twin			Non-twin			Twin		
	α	*M*	SD	α	*M*	SD	α	*M*	SD	α	*M*	SD
**Wave 1**												
Agency	0.80	−0.12	1.04	0.79	−0.11	1.01	0.79	0.12	0.94	0.81	0.13	0.97
Extraversion	0.78	0.07	1.00	0.77	0.04	0.98	0.77	−0.07	0.99	0.79	−0.05	1.02
Agreeableness	0.77	0.26	0.85	0.78	0.23	0.87	0.81	−0.28	1.07	0.81	−0.30	1.08
Conscientiousness	0.53	0.10	0.97	0.54	0.11	1.00	0.59	−0.11	1.01	0.55	−0.14	0.98
Neuroticism	0.76	0.11	1.02	0.76	0.07	0.99	0.73	−0.12	0.97	0.76	−0.08	1.00
Openness	0.78	−0.09	1.05	0.78	−0.04	1.03	0.77	0.09	0.94	0.77	0.05	0.96
Generativity	0.84	0.04	1.00	0.84	0.05	0.99	0.84	−0.04	1.00	0.85	−0.06	1.00

*Note.* Variables were standardized with respect to the grand mean and standard deviation at wave 1.

**Table 2:** Zero-order correlation matrix from the non-twin (upper triangle) and twin (lower triangle) MIDUS wave 1 samples.

	1.	2.	3.	4.	5.	6.	7.	8.	9.
1. Age	--	.104	− .008	− .031	.079	.053	− .144	− .078	− .012
*99% CI*	*(.065, .143)*	*(−.047, .031)*	*(−.047, .031)*	*(−.069, .008)*	*(.040, .117)*	*(.015, .092)*	*(−.182, − .106)*	*(−.116, − .039)*	*(−.051, .026)*
2. BMI	.092		− .005	− .040	.011	− .128	− .010	− .063	.035
*99% CI*	*(.030, .154)*	*--*	*(−.045, .034)*	*(−.079, .000)*	*(−.028, .051)*	*(−.167, − .089)*	*(−.050, .029)*	*(−.10, − .023)*	*(−.005, .074)*
3. Generativity	.021	− .028	-	**.390**	**.330**	.259	− .138	**.446**	**.358**
*99% CI*	*(−.041, .083)*	*(−.091, .036)*		*(.357, .423)*	*(.295, .365)*	*(.222, .295)*	*(−.176, − .099)*	*(.414, .476)*	*(.324, .392)*
4. Extraversion	.021	− .107	**.398**	--	**.529**	.268	− .114	**.515**	**.509**
*99% CI*	*(−.041, .082)*	*(−.169, − .044)*	*(.344, .449)*		*(.501, .557)*	*(.232, .304)*	*(−.182, − .106)*	*(.486, .543)*	*(.479, .537)*
5. Agreeableness	.055	− .054	**.337**	**.516**	--	.289	− .041	**.356**	.097
*99% CI*	*(−.007, .117)*	*(−.117, .009)*	*(.280, .391)*	*(.469, .560)*		*(.253, .324)*	*(−.080, − .003)*	*(.321, .389)*	*(.058, .135)*
6. Conscientious-ness	− .028	− .128	.234	.275	.262		− .187	.261	.239
*99% CI*	*(−.090, .034)*	*(−.190, − .065)*	*(.174, .293)*	*(.217, .332)*	*(.203, .319)*		*(−.225, − .150)*	*(.225, .297)*	*(.202, .276)*
7. Neuroticism	− .153	.049	− .117	− .172	− .038	− .219	--	− .149	− .083
*99% CI*	*(−.213, − .092)*	*(−.014, .112)*	*(−.178, − .054)*	*(−.231, − .110)*	*(−.100, .024)*	*(−.277, − .159)*		*(−.187, − .111)*	*(−.122, − .044)*
8. Openness to experience	− .055	− .086	**.432**	**.519**	**.346**	**.314**	− .193		**.516**
*99% CI*	*(−.117, .007)*	*(−.148, − .022)*	*(.379, .482)*	*(.471, .563)*	*(.290, .400)*	*(.256, .369)*	*(−.253, − .133)*		*(.487, .544)*
9. Agency	− .017	.008	**.349**	**.549**	.082	.252	− .106	**.514**	--
*99% CI*	*(−.079, .046)*	*(−.056, .071)*	*(.292, .403)*	*(.504, .592)*	*(.020, .144)*	*(.193, .310)*	*(−.167, − .044)*	*(.466, .559)*	

*Note.* Correlations greater than 0.30 are bolded.

**Table 3:** Multivariate regression results for associations between Agency and the Big Five factors in the non-twin and twin samples.

	Non-twin *β* se		Twin *β*se	
Extraversion	0.452	0.015	0.530	0.024
Agreeableness	−0.302	0.014	−0.330	0.021
Conscientiousness	0.114	0.012	0.097	0.020
Neuroticism	0.047	0.012	0.061	0.019
Openness to experience	0.373	0.014	0.339	0.022

**Table 4:** Model implied correlation matrix from the correlated latent growth models in the non-twin (left) and twin (right) samples.

	Non-twins		Twins	
	Agency Slope	Agency Intercept	Agency Slope	Agency Intercept
1. Extraversion				
Intercept	−0.263	0.640	−0.296	0.711
Slope	0.896	−0.173	0.939	−0.308
2. Agreeableness				
Intercept	−0.065	0.140	−0.126	0.124
Slope	0.486	−0.153	0.399	−0.161
3. Conscientious				
Intercept	−0.105	0.301	−0.165	0.373
Slope	0.503	−0.140	0.962	−0.358
4. Neuroticism				
Intercept	0.022	−0.111	0.035	−0.160
Slope	0.014	−0.047	−0.204	0.086
5. Openness				
Intercept	−0.287	0.654	−0.402	0.679
Slope	0.934	−0.213	1.00	−0.282
6. Generativity				
Intercept	−0.074	0.475	−0.181	0.469
Slope	0.346	−0.037	0.441	−0.136

**Table 5:** The correlated genetic and environmental influences on growth to the genetic and environmental influences on stability parameters from bivariate longitudinal models with agency. A refers to genetic influences; C refers to shared environment; and, E refers to unique environment.

	Agency Intercept			Agency Slope		
	*r*A	*r*C	*r*E	*r*A	*r*C	*r*E
Extraversion						
Intercept	.661	--	.464	− .230	--	.053
Slope	− .037	--	− .234	.527	--	.315
Agreeable						
Intercept	.131	.066	.063	− .053	− .044	− .030
Slope	− .033	− .019	− .080	.085	.104	.183
Conscientious						
Intercept	.302	--	− .194	.132	.019	− .170
Slope	--	.002	.161	--	− .082	− .174
Neuroticism						
Intercept	− .138	--	.057	.082	--	− .105
Slope	.133	--	.023	− .040	--	− .140
Openness to experience						
Intercept	.589	--	− .450	− .276	--	.144
Slope		--	--		--	--
Generativity						
Intercept	.416	--	.316	− .192	--	− .114
Slope	--	--	.302	--	.482	− .055

## Data Availability

Data from the MIDUS study is available to the public through ICSPR (http://midus.wisc.edu/data/index.php). We do not provide cleaned data to encourage downloading directly from MIDUS and using the scripts provided to recreate the results. MIDUS Milwaukee sample is protected behind a request for access.
